# First Radiologic Description of CIC-FOXO4 Sarcoma: A Case Report of a Rare and Aggressive Sarcoma and Review of the Literature

**DOI:** 10.7759/cureus.97330

**Published:** 2025-11-20

**Authors:** Ayoub Nahal

**Affiliations:** 1 Pathology and Laboratory Medicine Institute, Cleveland Clinic Abu Dhabi, Abu Dhabi, ARE

**Keywords:** cic-foxo4 sarcoma, cic-rearranged sarcoma, epithelioid histology, fusion sarcoma, molecular diagnosis

## Abstract

CIC-rearranged sarcomas are a recently recognized subset of undifferentiated round cell sarcomas, distinct from Ewing sarcoma in both clinical behavior and molecular profile. While CIC-DUX4 is the most described fusion, CIC-FOXO4 is exceedingly rare, with only a handful of reported cases. We report a 47-year-old male with a 3.6 cm intramuscular mass in the left lateral abdominal wall. Molecular testing confirmed a CIC-FOXO4 sarcoma. The lesion was surgically excised and showed predominant epithelioid morphology within a desmoplastic fibrous stroma. The patient is alive with disease, developing three metastatic pulmonary nodules six months after initial presentation, for which he received adjuvant chemotherapy. To our knowledge, this represents the sixth reported case of CIC-FOXO4 sarcoma in the English literature and the first with radiologic description. This case adds to the limited radiologic literature on this rare entity and emphasizes the importance of including CIC-rearranged sarcoma in the differential diagnosis of atypical soft tissue masses.

## Introduction

Undifferentiated round cell sarcomas lacking EWSR1 rearrangements have emerged as a genetically and clinically distinct entity from Ewing sarcoma. Among these, CIC-rearranged sarcomas, particularly CIC-DUX4 fusion tumors, represent the most common subtype and have been formally recognized in the 2020 WHO Classification of Soft Tissue and Bone Tumors [1]. These tumors most commonly affect adolescents and young adults, typically arising in soft tissue, and display an aggressive clinical course with limited response to chemotherapy. They demonstrate a wide spectrum of morphology, including round, epithelioid, and spindle cells [2]. CIC-FOXO4 is a rare and less well-characterized fusion variant in this category, with only a handful of cases reported [3-7]. We describe the radiology and pathology of a rare case of CIC-FOXO4 sarcoma arising from the abdominal wall musculature of a middle-aged man and provide a comprehensive review of the literature. This study was approved by the institutional review board, and the requirement for informed consent was waived.

## Case presentation

A 47-year-old male presented with a painless swelling in the left lower abdominal region, which had been gradually increasing in size over two months. Physical examination revealed a firm, non-tender, and non-mobile mass in the left lateral abdominal wall.

Imaging

**Figure 1:**
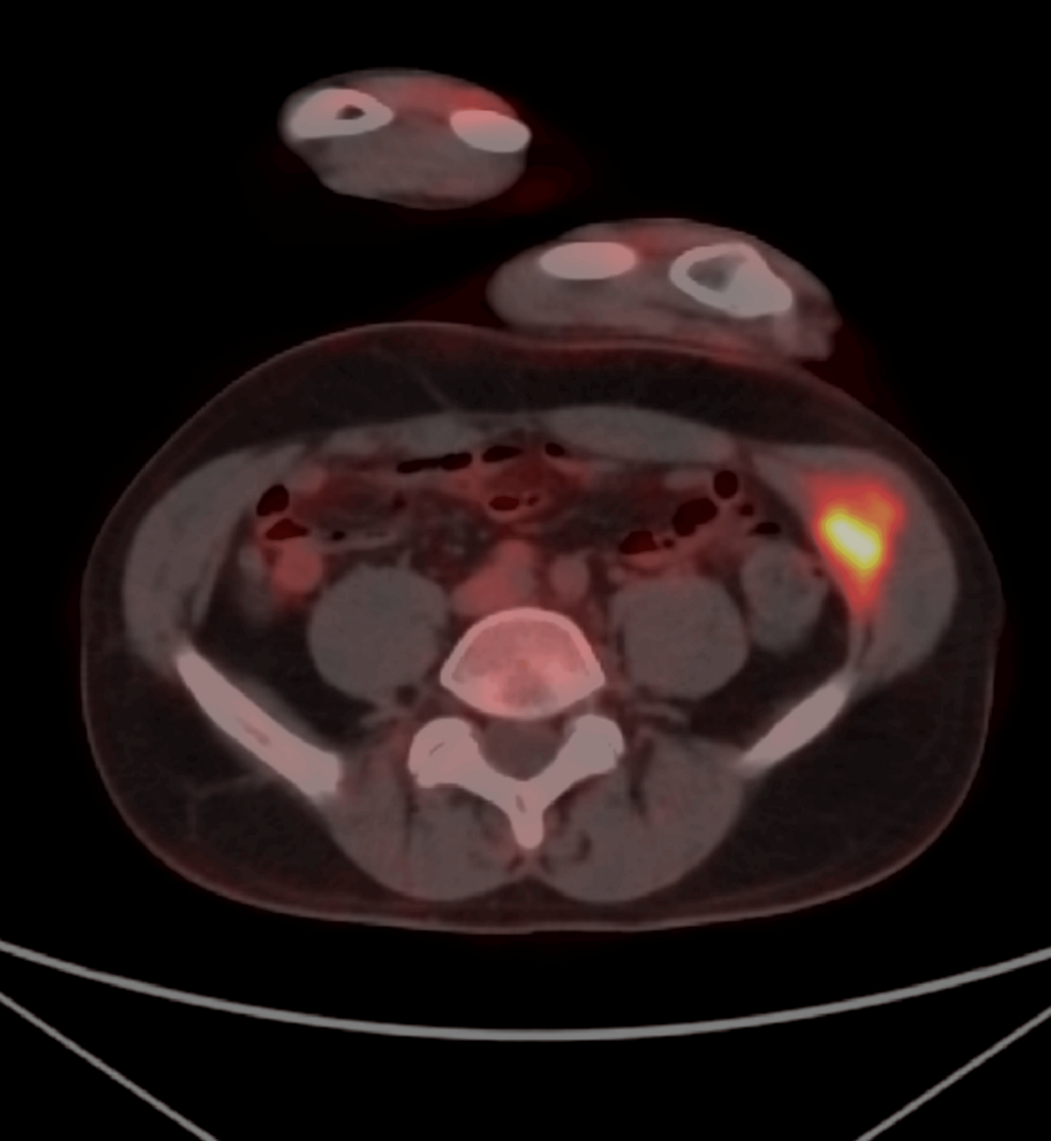
Positron emission tomography (PET)-computed tomography (CT) scan Asymmetry in size between the left and right internal oblique muscle of the abdominal wall due to a mass showing abnormal fluorodeoxyglucose (FDG) activity in the left.

**Figure 2:**
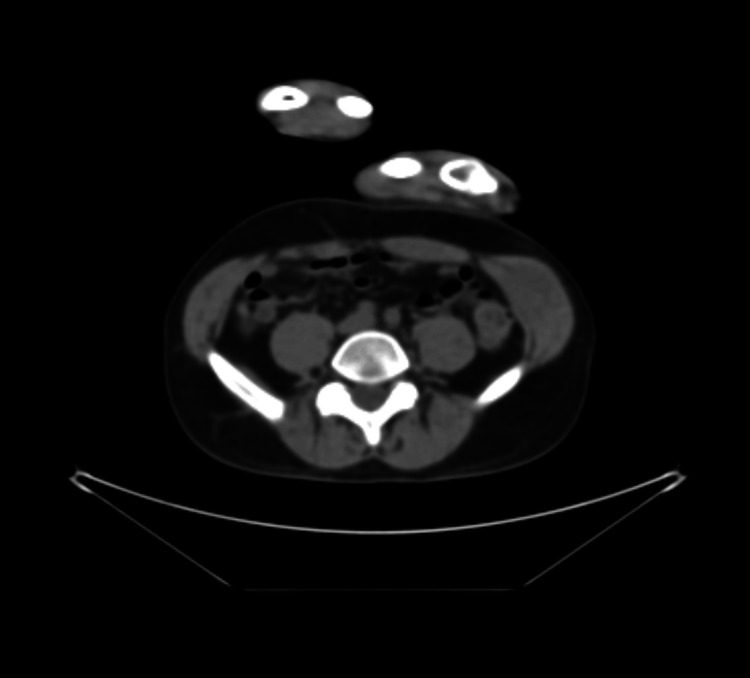
CT scan of the pelvis Asymmetry in size between the left and right internal oblique muscles of the abdominal wall due to an intramuscular mass in the left.

**Figure 3:**
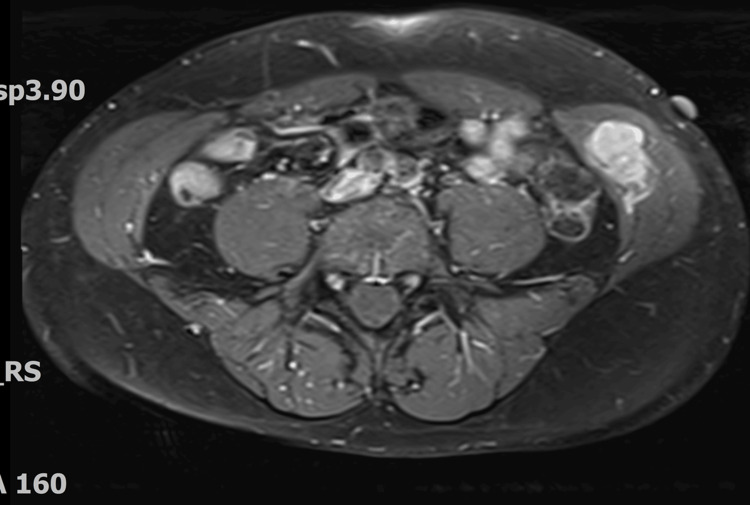
MRI post-contrast T1 with FAT-SAT (fat-saturation) Space-occupying mass with enhancement measuring 3.0 cm in the greatest dimension occupying the left Internal oblique muscle.

**Figure 4:**
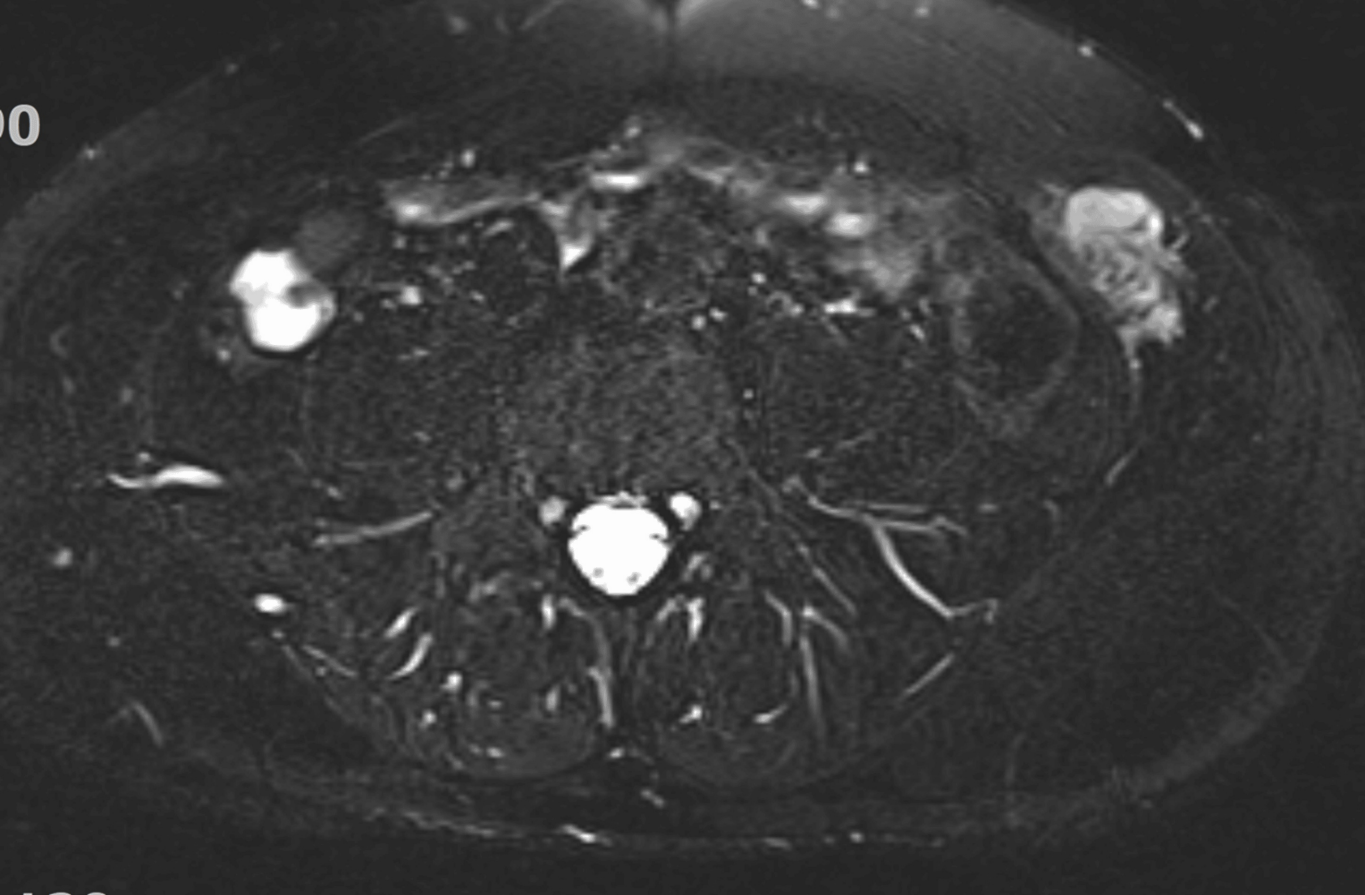
MRI axial T2 FAT SAT (fat-saturation) Heterogeneous hyperintensity T2 space occupying mass in the left internal oblique muscle.

**Figure 5:**
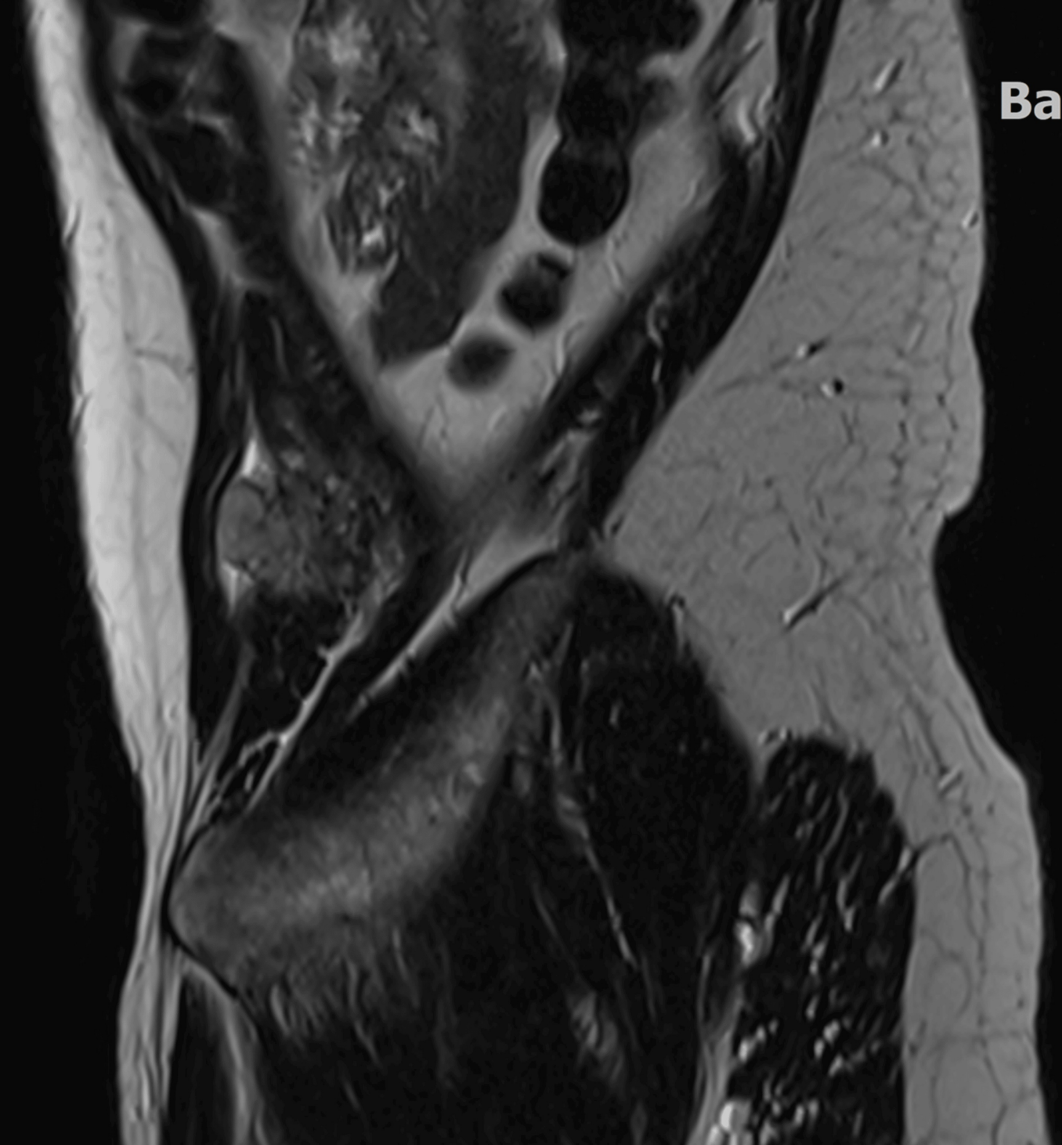
MRI sagittal T2 without FAT SAT (fat-saturation) Heterogeneous hyperintensity T2 space occupying the mass in the left internal oblique muscle.

An ultrasound of the abdominal wall revealed a 3.6 × 2.5 × 1.9 cm heterogeneous hypoechoic lobulated mass within the left internal oblique muscle with internal vascularity. A PET-CT scan revealed asymmetry between the left and right internal oblique muscles of the abdominal wall, with a 3.6 cm intramuscular mass exhibiting increased FDG uptake (Figure 1). Contrast-enhanced CT of the pelvis confirmed a space-occupying lesion within the left internal oblique muscle (Figure 2). Subsequent MRI of the abdomen with IV contrast demonstrated a lobulated soft tissue mass measuring 2.6 cm (CC) × 3.6 cm (transverse) × 2.0 cm (AP) within the left internal oblique muscle, with extension into the external oblique and transversus abdominis muscles. The mass was isointense to slightly hyperintense on T1-weighted images, heterogeneously hyperintense on T2 and T2 fat-suppressed sequences, and showed progressive post-contrast enhancement (Figures 3-5). Radiologic differential diagnosis included sarcoma, desmoid-type fibromatosis, and vascular malformation.

Histopathology and molecular testing

**Figure 6:**
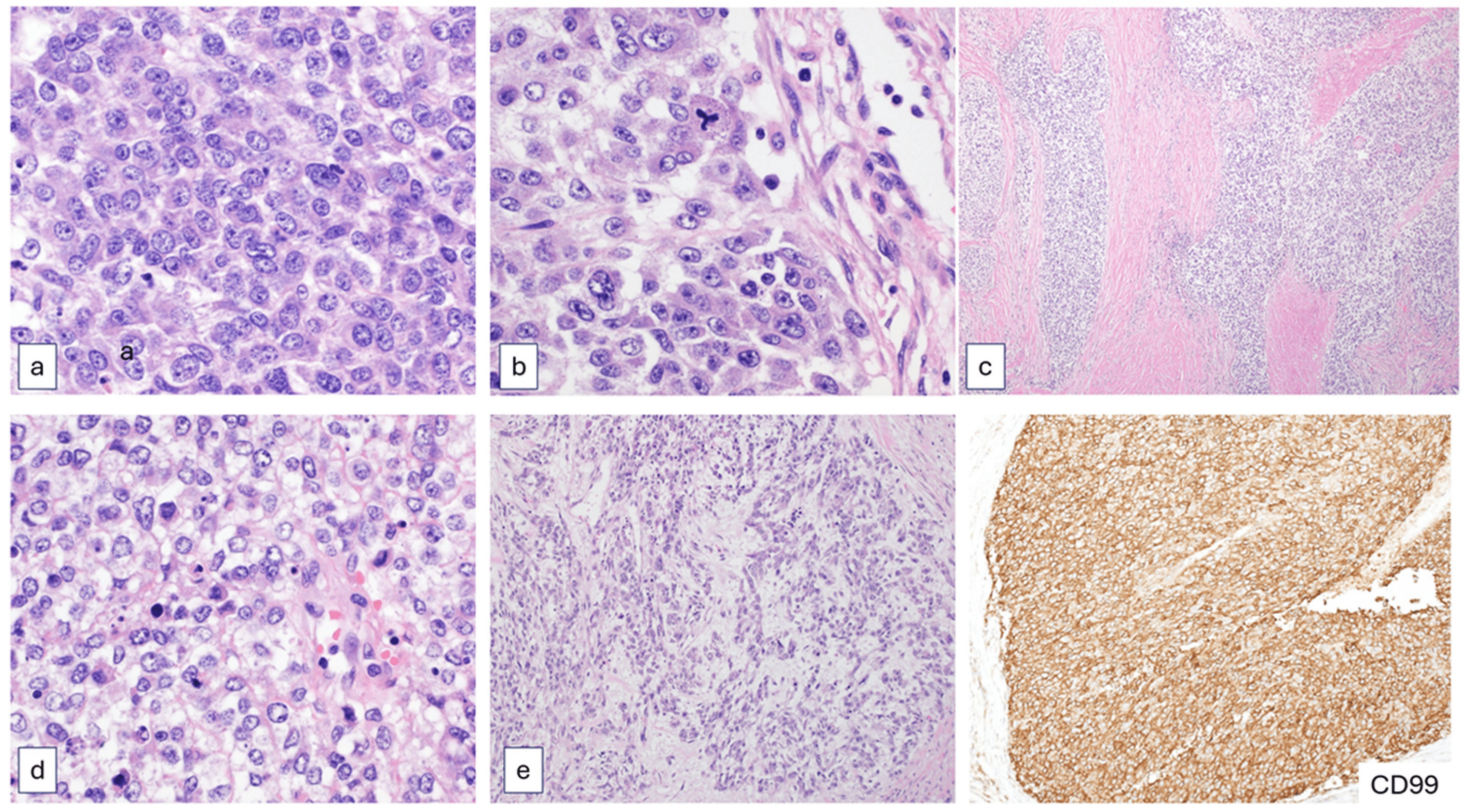
Histologic appearance of the tumor a: Sheets of undifferentiated round cells with enlarged nuclei. b: Epithelioid morphology is present with pleomorphism and atypical mitosis. c: Desmoplastic fibrous stroma dissecting tumor nodules with focal necrosis. D: Clear cell morphology with karyorrhectic debris. e: Mixed spindle and epithelioid morphology with myxoid stromal background. CD99 (EPR 3097Y, cell marque): strong and diffuse membranous expression similar to classic Ewing sarcoma. (hematoxylin and eosin stain. a, b, d: 400x magnification. c: 40x magnification. e: 100x magnification).

**Figure 7:**
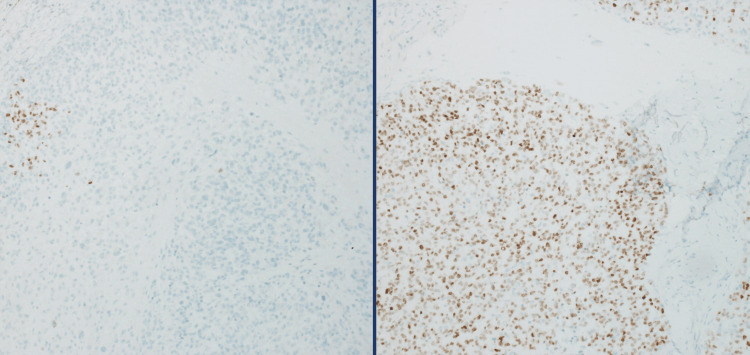
NKX2.2 (clone EP336; cell marque) Variable expression. Patchy positive (10% of the tumor nuclei).

The mass was initially biopsied and revealed a necrotic, mitotically active, malignant tumor composed of undifferentiated round to spindle cells embedded in a desmoplastic stroma, with areas showing sheets of large epithelioid and pleomorphic cells. The immunohistochemical profile was only significant for CD99 and WT-1 expression. The diagnosis of undifferentiated sarcoma was rendered with the suspicion of a possible fusion-type sarcoma. The tumor was excised and revealed a similar morphology with high-grade epithelioid-rich pleomorphic neoplasm with areas of necrosis and brisk mitotic activity. Immunohistochemically, the tumor was diffusely positive for CD99, while focally positive for both WT-1 and NKX2.2 (Figures 6, 7). All other stains, including S100, SOX10, GFAP, EMA, CAM5.2, CKAE1.3, CD34, ERG, Desmin, Myogenin, Melan-A, CKIT, and CK19, were negative. INI-1 expression was retained. Given the suspicion of fusion-type sarcoma, RNA-based next-generation sequencing was performed, which identified a fusion product between CIC exon 20 (NM_015125.4) and FOXO4 exon 2 (NM_005938.4). These findings confirmed the diagnosis of CIC-FOXO4 sarcoma.

## Discussion

CIC-rearranged sarcomas are a distinct subset of undifferentiated small round cell tumors. Unlike Ewing sarcomas, these tumors lack EWSR1 fusions and have a significantly more aggressive clinical behavior. The most common rearrangement involves CIC-DUX4, while alternate fusions, such as CIC-FOXO4, are extremely rare and less well-characterized. To our knowledge, only five cases have been documented in the literature with molecular confirmation.

The landmark study of 115 cases of CIC-rearranged sarcomas was published by Antonescu et al. [2] in 2017. They were described as aggressive tumors arising in the soft tissues of children and young adults. All cases showed a CIC gene break-apart signal. CIC-DUX4 fusion was detected in 57% of cases, with either DUX4 on 4q35 (35%) or on 10q26 in (22%) cases. No FOXO4 gene rearrangements were present in 39 cases tested, and 76 cases (43%) had an unknown molecular signature. Distinctive features were associated with CIC-DUX4-positive tumors, most importantly, a wider spectrum of cytomorphology, with a mixture of round, spindle, and epithelioid cells. Eighty-four percent of cases showed variable expression of CD99, and only 23% a diffuse pattern. Nuclear WT1 reactivity was also a consistent finding.

Prior to Antonescu’s formal description of CIC-rearranged sarcomas, Yustein et al. described in 2010 a case of small round cell tumor of the abdomen in a 10-year-old male, raising suspicion for a desmoplastic small round cell tumor [3]. Molecular studies and reverse transcription polymerase chain reaction (RT-PCR) failed to identify the EWS-WT1 translocation associated with desmoplastic small round cell tumors. Cytogenetic analysis found a novel translocation (X;19) (q13;13.3), which has not been reported in small round cell tumors of childhood or adults. The transcription factor FOXO4, which was located on Xq13, was known to have a role in apoptosis. Although reported as an undifferentiated small round cell tumor, their case was likely to represent the first case of CIC-FOXO4 sarcoma [3]. The tumor involved the liver, colon, and right hemidiaphragm. The patient underwent surgery and a complete gross excision. Imaging studies did not reveal distant metastatic disease.

Sugita et al. described in 2014 the second case of CIC-FOXO4 sarcoma in a 63-year-old male with a 3 cm intramuscular mass in his right posterior neck [4]. Undifferentiated round cells embedded in a desmoplastic fibrous stroma were the most characteristic histology and raised the suspicion of desmoplastic round cell tumor (DSRCT). High-throughput transcriptome sequencing revealed a gene fusion, and the genomic rearrangement between the CIC and FOXO4 genes was identified by fluorescence in situ hybridization. Similar to the previous case, no metastatic disease was seen, and therefore the patient underwent surgery followed by chemo-radiation. At a six-month follow-up, the patient had no evidence of disease.

The third case, published by Solomon et al., was described in a 13-year-old boy presenting with a mass on his posterior scalp [5]. The tumor morphology was of undifferentiated round cells lacking a desmoplastic stroma. The patient had an aggressive clinical course with both local recurrence and lung metastasis.

The fourth case was described by Connolly et al. in 2022 [6]. With the aim of further characterising the clinical features of CIC-rearranged sarcomas and exploring clinical management, including systemic treatments and outcomes, a cohort study of 18 patients diagnosed between 2014 and 2019 was analysed. Only one of 18 patients showed a CIC-FOXO4 fusion, from a 27-year-old male with a 6.0 cm thigh mass. The histologic features of this sarcoma were not provided, but the patient received surgery, adjuvant chemo-radiation. At the time of publication, the patient was alive with lung disease.

The aggressiveness of CIC-FOXO4 sarcoma was also underscored in the most recent report by Babkoff et al. (2023), who described the fifth case in a 46-year-old female presenting with a 7 cm rapidly growing scalp lesion with bone involvement. Microscopic examination revealed a highly cellular neoplasm with immature round cells and rhabdoid-like morphology as well. The immunohistochemical profile was not diagnostic. Next-generation sequencing (FoundationOne Heme© assay) revealed a CIC-FOXO4 fusion. The patient had a rapid local recurrence and intractable systemic spread, including brain metastases. He received multiple cycles of chemoradiation but died of disease after three years of initial presentation [7].

We have found no radiologic description of CIC-FOXO4 sarcoma in the literature, including the above-referenced articles [2-7]. The only published paper by Brady et al. describing the radiology of CIC-DUX4 sarcomas analyzed 12 pathologically confirmed cases, with the majority (83%) arising in soft tissue and the remainder in bone or lung [8]. Imaging review (MRI, CT, and PET) revealed that these tumors typically present as well-defined, heterogeneously enhancing masses with frequent necrosis, perilesional edema, and prominent vascularity on MRI. Additional features included hemorrhage, fluid levels, and occasional bone erosion on CT, while PET scans demonstrated FDG-avid lesions with high SUVmax values, with a mean SUVmax of 13.2.

Our case adds valuable insight to the limited literature on CIC-FOXO4 sarcomas. It stands out for several reasons: first, the patient was middle-aged, while most reported cases involve adolescents and young adults. Second, to our knowledge, this is the first case in the literature with a detailed radiologic description. Thirdly, the tumor’s morphology did not resemble the typical small round cell sarcoma, despite strong CD99 expression and patchy NKX2.2 positivity.

Importantly, while NKX2.2 has recently emerged as a highly specific marker for Ewing sarcoma [9], usually showing intense, diffuse nuclear expression, it can also be focally and patchily expressed, thereby reducing its specificity. Such patchy expression has been observed in various other malignancies [10], highlighting the need for caution in interpreting NKX2.2 staining as definitive evidence of Ewing Sarcoma.

Given the limited treatment response to existing chemotherapy regimens, surgery remains the cornerstone of treatment when feasible. Chemotherapy is often given despite poor efficacy due to the lack of alternative options. Long-term outcomes remain guarded, and molecular profiling is essential for diagnosis, prognosis, and potential inclusion in clinical trials.

## Conclusions

CIC-FOXO4 sarcoma is a rare and highly aggressive subset of CIC-rearranged sarcomas. It is notable for its morphologic diversity, including the frequent, but not consistent, presence of epithelioid to rhabdoid morphology and desmoplastic stroma, raising a differential diagnosis of DSRCT and other epithelioid-rich malignancies. Its imaging characteristics, while variable, consistently point towards an aggressive tumor, underscoring the importance of considering CIC-rearranged sarcomas in the radiologic differential diagnosis of aggressive soft tissue tumors. Lastly, definitive diagnosis requires molecular analysis, reinforcing the importance of considering rare translocations in round cell sarcomas.
